# Stressors related to the COVID-19 pandemic and their association with distress, depressive, and anxiety symptoms in cancer out-patients

**DOI:** 10.3389/fpsyg.2023.1100236

**Published:** 2023-06-02

**Authors:** Tamara Frank, Theresia Pichler, Sabrina Maier, Ineke Batenhorst, Tanja Abawi, Nadia Harbeck, Hana Algül, Volker Heinemann, Kerstin Hermelink, Friederike Mumm, Andreas Dinkel

**Affiliations:** ^1^Comprehensive Cancer Center Munich, Munich, Germany; ^2^Department of Psychosomatic Medicine and Psychotherapy, Klinikum rechts der Isar, School of Medicine, Technical University of Munich, Munich, Germany; ^3^Department of Obstetrics and Gynecology, LMU University Hospital, Munich, Germany; ^4^Mildred-Scheel-Professor of Tumor Metabolism, Technical University of Munich, Munich, Germany; ^5^Department of Internal Medicine III, LMU University Hospital, Munich, Germany

**Keywords:** COVID-19, coronavirus, stressors, cancer, distress, depression, anxiety, somatic symptoms

## Abstract

Patients with cancer might be particularly prone to stress caused by the COVID-19 pandemic. The aim of this study was to investigate the impact of pandemic-related stressors on oncological patients’ psychological well-being. During the second wave of the COVID-19 pandemic in Germany 122 cancer out-patients of the Comprehensive Cancer Center Munich reported on COVID-19-related stressors (information satisfaction, threat perception, and fear of disease deterioration) and answered standardized questionnaires for psychosocial distress (DT) as well as depression and anxiety symptoms (PHQ-2, GAD-2). Multiple linear regression analyses were used to identify associations of the COVID-19-related stressors with psychological symptoms, controlling for sociodemographic, psychological (self-efficacy, ASKU) and clinical (somatic symptom burden, SSS-8) variables. Initially, satisfaction with information was significantly negatively associated with all three outcome variables. Fear of disease deterioration was associated with distress and depressive symptoms. After controlling for additional variables, only satisfaction with information remained an independent determinant of anxiety (*β* = −0.35, *p* < 0.001). All three outcomes were most strongly determined by somatic symptom burden (*β* ≥ 0.40, *p* < 0.001). The results of this study tentatively suggest that physical well-being overrides the relevance of some COVID-19-related stressors for oncological patients’ psychological wellbeing. Physical symptoms are strongly tied to personal wellbeing as they are associated with suffering from cancer, which might be more central to personal wellbeing than the possibility of getting infected with SARS-CoV-2. However, satisfaction with the information received seems to be important beyond physical wellbeing, as this emerged as an independent determinant of anxiety.

## 1. Introduction

The COVID-19 pandemic and the necessary protective measures have had effects on the population’s mental health leading to higher levels of stress, anxiety and depression (Peters A. et al., 2020). Even before the pandemic, cancer patients displayed an increased risk of several common mental disorders (Lu et al., 2016) and have been found to show higher rates of symptoms of depression and anxiety as compared to the general population (Hinz et al., 2010; Hartung et al., 2017). Besides, many cancer patients are affected by cancer-related emotional burden like distress (Herschbach et al., 2020), demoralization (Vehling et al., 2017) and fear of progression (Dinkel et al., 2021b).

Cancer patients might be particularly prone to stress caused by the pandemic situation like the threat of getting infected and the measures against viral spread (Lou et al., 2020; Büssing et al., 2021). Due to physical vulnerability like immunosuppression, many cancer patients have an increased risk for infection and COVID-19 associated treatment outcomes like a severe course of their disease or thromboembolic events (Palaskas et al., 2020; Zhang et al., 2020). Consequently, cancer patients might experience an even greater pandemic-related threat perception.

Besides pre-existing stressors and higher infection-related risks, many patients with cancer have been affected by pandemic induced structural changes in the health care system and limited medical resources due to their dependency on close check-ups and treatment appointments (Chen et al., 2020; Fröhling and Arndt, 2020). Emergency care and treatment of COVID-19 patients had been prioritized, which led to changes and adaptations or postponements in existing treatment plans (Tougeron et al., 2021). These (possible) changes to patients’ cancer care or recovery could cause additional insecurity and lead to increased worries about impaired treatment quality and effectiveness, or about a deteriorating health condition (Ludwigson et al., 2022).

Pandemic-related information transfer poses another possible source of stress. The ubiquitous pandemic-induced uncertainties were increased by patients’ diseases and potential impacts on their treatment. For example, for many cancer patients conflicting public media reports about the danger and the course of SARS-CoV-2-infection caused confusion and the need for adequate information and reliable resources (Büssing et al., 2021). Discussing these concerns and discrepancies with their physician could help reduce patients’ confusion and increase acceptance, if patients are satisfied with the communication (Sokas et al., 2021). However, opportunities for patient-doctor communication and consultations were often limited.

Several studies found that cancer patients’ psychological health was greatly affected by the COVID-19 pandemic (Momenimovahed et al., 2021). For example, French cancer patients experienced a significant number of COVID-19-related stressors, which correlated with higher levels of anxiety, depressive symptoms and insomnia (Massicotte et al., 2021). High rates of emotional distress were also found among Spanish cancer patients (Toquero et al., 2021). And Bäuerle et al. (2021) detected an increase in depression and anxiety symptoms and distress in German patients during the first pandemic wave. However, most of these studies controlled for a limited number of known risk factors for psychological distress in patients with cancer. Especially, somatic symptom burden has not been treated as a control variable in these studies. So, it remains unclear whether COVID-19-related stressors show an independent association with psychological distress. Moreover, data on the impact of different psychosocial determinants on cancer out-patients during the second pandemic wave in Germany is scarce.

To address this, we conducted a survey among out-patient cancer patients of the Comprehensive Cancer Center Munich between November 2020 and February 2021 to identify information satisfaction, threat perception and fear of disease deterioration as COVID-19-related stressors and their potential associations to psychological distress.

## 2. Method

### 2.1. Design

This cross-sectional study used a consecutively gathered sample of cancer patients receiving out-patient treatment at the Comprehensive Cancer Center Munich within a pre-defined period of time (Pichler et al., 2022a). Members of the study team approached patients within the context of their treatment appointments and patients who were interested in participation were given the participant information and declaration of consent form. Patients qualified for study participation if they had an established cancer diagnosis and were at least 18 years old. Exclusion criteria were stable, recurrence-free disease for more than 10 years, known psychiatric diagnosis, a lack of ability to consent to participation and insufficient German language skills. Patients who consented to participate were assessed via the phone. The standardized self-reporting questionnaires and the newly developed items were administered by telephone to allow for personal contact to patients without risking infection.

### 2.2. Ethics statement

The study was developed in accordance with the fundamental principles of Good Clinical Practice established in the Declaration of Helsinki. It was approved by local ethics committees (LMU Hospital: 20-615, Klinikum rechts der Isar: 390/20 S). All participants provided written informed consent to participate in this study.

### 2.3. Instruments

A questionnaire covering COVID-19-related stressors was developed via expert consensus and consolidated and revised within a multi-level process. Items covered knowledge, information needs, risk and threat perception, COVID-19-related worries and willingness to be vaccinated. Items were regarded as consented if the expert group (AD, FM, TF and TP) agreed that the item was comprehensible and content-valid. Most items were assessed on a five-point Likert scale, including the three items on COVID-19-related stressors (Pichler et al., 2022a). Psychosocial distress, symptoms of anxiety and depression, somatic symptoms, and self-efficacy were assessed via standardized self-reporting questionnaires. All measures have already been used in German samples and have prooved reliable and valid.

#### 2.3.1. COVID-19-related stressors

Satisfaction with information received was assessed using the item “How satisfied are you with the information you received from your oncological team regarding the impact of a COVID-19 infection on your disease?” The response options on the five-point Likert scale ranged from “very dissatisfied” (1) to very satisfied (5).

To measure threat perception the item “How threatening do you regard a COVID-19 infection would be for yourself?” was used. The response options were “not at all” (1) to “very much” (5).

For fear of disease deterioration, the question “How concerned are you that your cancer would worsen because of possible changes in treatment plans during the COVID-19 pandemic?” was asked. Here, again, the possible responses ranged from “not at all” (1) to “very much” (5).

#### 2.3.2. Psychosocial distress

For cancer-related distress, the NCCN distress thermometer was used (DT) (Mehnert et al., 2006). The DT is a one-item, 11-point Likert scale, visually represented as a thermometer that ranges from 0 (no distress) to 10 (extreme distress) and measures patients’ level of distress over the course of the week prior to assessment. We used the cut-off score ≥ 5 to indicate a clinical level of psychosocial distress (Mehnert et al., 2006; Peters L. et al., 2020).

#### 2.3.3. Symptoms of anxiety and depression

Short form scales of the Patient Health Questionnaire (Löwe et al., 2010) were used to measure symptoms of depression (PHQ-2; three items) and anxiety (GAD-2; three items). Each item is answered on a Likert scale ranging from 0 to 3. For evaluation the scores on both scales are summed up. A summed score of ≥3 is considered an indicator for pathological depression and anxiety, respectively. In the present study, Cronbach’s α indices were acceptable for both, the PHQ-2 (α = 0.77) and the GAD-2 (α = 0.70).

Somatic symptom burden was assessed with the Somatic Symptom Scale-8 (SSS-8) (Gierk et al., 2014). The SSS-8 measures the somatic symptom burden over the course of the last week. The burden of eight typical somatic symptoms is rated on a Likert scale ranging from 0 to 4. The scores are summed up to build a total score. In the present study, the internal consistency for the full scale was α = 0.82.

#### 2.3.4. Self-efficacy

The General Self-Efficacy Short Scale (in German: ASKU) was used to measure self-efficacy via three items, which are answered on a Likert scale ranging from 1 to 5 (Beierlein et al., 2013). The scores on these three items are summed up, higher scores indicating higher self-efficacy. In the present study, internal consistency for this scale was good (α = 0.85).

#### 2.3.5. Sociodemographic and clinical characteristics

Additionally, patients answered questions about sociodemographic (gender, age, marital status, children, single parent, living situation, education, employment, economic situation) and medical (diagnosis, disease condition, illness duration, metastases, current treatment, general health status) variables. Complementing details regarding diagnosis and treatment were taken from the clinical documentationsystems.

### 2.4. Statistical analyses

Statistical analyses were conducted using IBM SPSS 26. Mean values, standard deviations, and frequencies are reported for sociodemographic and clinical variables. Group differences for patients above and under the cut-off (dichotomized for distress, symptoms of depression and anxiety) were performed by means of t-tests for independent samples. Independent variables included COVID-19-related variables (satisfaction with information, threat perception, and fear of disease deterioration). Effect sizes (Cohen’s d) of between-group differences are also reported. Additionally, we performed three multiple linear regression analyses to identify variables independently associated with distress, symptoms of depression, and symptoms of anxiety. For each outcome, two models were performed. Model 1 included the COVID-19-related predictors: satisfaction with information, threat perception, and fear of disease deterioration for each. Model 2 controlled for the effects of age, sex, current treatment (dichotomized/dummy-coded to “Yes” and “No”), previous or current psychosocial treatment (dichotomized/dummy-coded to “Yes” and “No”), self-efficacy, and somatic symptoms. All statistical tests were two-tailed. The proportion of missing values in the present sample was negligible due to the face-to-face or telephone interviews. Therefore, missing values were not replaced for the analysis. We calculated intercorrelations and found adequate inflation factors (VIF) between the predictors.

### 2.5. Context

Between 11/2020 and 02/2021 122 out-patient cancer patients of the Comprehensive Cancer Center Munich participated in the study after giving informed consent.

## 3. Results

### 3.1. Study population

The study population comprised 122 cancer out-patients with different tumor entities from the two university hospitals of the Comprehensive Cancer Center Munich. Sixty-eight (55.7%) participants were female; mean age was 58.5 years (*SD* = 14.5; Range: 23 to 87 years). Frequent cancer diagnoses were hematological (24.6%, *n* = 30), gastrointestinal (20.5%, *n* = 25), and breast cancer (19.7%, *n* = 24). Most patients were currently undergoing chemotherapy (43%, *n* = 53), whereas 27% (*n* = 33) did not receive any therapy at the time of data collection. For further details of the study population, please see Table 1.

**Table 1:** Sociodemographic and clinical variables of the study population (*n* = 122).

Sociodemographic variables		
	*M*	*SD*
Age	58.5	14.5
	***n***	***%***
Sex		
Female	68	55.7
Male	54	44.3
Age group		
≤50	36	29.5
51 to 65	43	35.2
66 to 75	28	23.0
76 and older	15	12.3
Marital status		
Single	21	17.2
Registered partnership/married	78	63.9
divorced/separated	17	13.9
Widowed	6	4.9
Educational level		
Elementary school/secondary school	15	12.3
Junior high/vocational school	36	29.5
High school	14	11.5
Graduated	52	42.6
Other/none	5	4.1
Children		
Yes	91	74.6
None	31	25.4
Employment		
Employed full-time/part-time	58	47.5
unemployed	–	–
Homemaker	3	2.5
Retired	53	43.4
Other	7	5.7
Subjective economic situation		
Very good	26	21.3
Good	56	45.9
Satisfactory	30	24.6
Not very good	7	5.7
Poor	3	2.5
**Clinical variables**		
Tumor entity		
Gastrointestinal	25	20.5
Urogenital	9	7.4
Gynecological	13	10.7
Breast	24	19.7
Head/Neck	3	2.5
Skin	3	2.5
Bone/Soft tissues	3	2.5
Endocrine tumors	2	1.6
Hämatological	30	24.6
Others	10	8.2
Disease status		
First Occurrence	79	64.8
Recurrence	20	16.4
Second tumor/third tumor	8	6.6
Remission	14	11.5
Unknown	1	0.8
Metastases		
Yes	53	43.8
No	61	50.4
Unknown	7	5.8
Illness duration		
Up to 3 months	12	9.8
4 to 12 months	33	27.0
More than 1 y and up to 5 y	44	36.1
More than 5 y	33	27.0
Current treatment (last month, multiple responses)		
Surgery	2	1.6
Chemotherapy	53	43.4
Radiotherapy	6	4.9
Hormonal therapy	8	6.6
Immunotherapie	11	9.0
Targeted therapy/antibody therapy	17	13.9
Other therapy	8	6.6
No therapy	33	27.0
Self-rated health		
Excellent	3	2.5
Very good	20	16.4
Good	56	45.9
Fair	35	28.7
Poor	8	6.6
Psychological treatment in the past		
Yes, uptake of psychiatric or psychotherapeutic treatment	22	18.0
Yes, uptake of psycho-oncological treatment	29	23.8
None	71	58.2

### 3.2. Descriptives

#### 3.2.1. Distress, depressive symptoms, anxiety, somatic symptoms

The results showed that 34.7% (*n* = 42/121; *M* = 3.7, *SD* = 2.4) of the participants had elevated levels of psychosocial distress, whereas 14.2% (*n* = 17/121) had elevated depressive symptoms (Cut-off ≥3; *M* = 1.3, *SD* = 1.4) and 15.6% (*n* = 19/122; *M* = 1.3, *SD* = 1.4) reported clinical anxiety. About 24.8% (*n* = 30/121) showed high to very high levels regarding somatic symptoms (Cut-off ≥ 12; *M* = 7.5, *SD* = 5.4).

#### 3.2.2. Information satisfaction, threat perception, and fear of disease deterioration

In this sample, 17.2% (*n* = 21) of the patients reported changes in current or planned cancer treatment. These changes mostly comprised postponed or canceled treatment appointments, postponed check-ups/other examination appointments or postponements due to a patient’s COVID-19 infection. 63.9% (*n* = 71/111) were rather or very satisfied with the information received from their oncologists, 21.6% (24) were undecided and 14.4% (*n* = 16/111) were quite or very unsatisfied. For 47.1% (*n* = 57/121), a potential infection with the coronavirus was rated as quite or very threatening, for 33.1% (*n* = 40) as medium threatening and for 19.9% (*n* = 24) as not at all or rather not threatening. 64.8% (*n* = 79/122) were not at all or rather not concerned that their cancer would worsen because of possible changes in treatment plans due to the pandemic. 16.4% (*n* = 20) were rather or very concerned and 18.9% (*n* = 23) chose the middle category.

### 3.3. Group differences regarding distress and symptoms of depression and anxiety

Independent *t*-tests were conducted to compare groups of high vs. low psychosocial burden (distress: cut-off ≥5; depression: cut-off ≥3; and anxiety symptoms: cut-off ≥3) for all three COVID-19-related variables (satisfaction with information, threat perception and fear of disease deterioration). Clinically distressed participants were less satisfied with information they received (*M* = 3.89, *SD* = 0.97) than non-distressed participants (*M* = 3.35, *SD* = 1.14; *t* (108) = 2.61, *p* = 0.010), see Table 2. Threat perception was higher in patients with symptoms of depression (*M* = 4.31, *SD* = 0.95) than in patients without depression symptoms (*M* = 3.35, *SD* = 1.05; *t* (117) = −3.44, *p* = 0.001), see Table 3. None of the comparisons between participants with or without anxiety symptoms reached significance. Results are depicted in Table 4.

**Table 2:** Group differences for patients above and below the cut-off for distress (DT).

Items	DT < 5		DT ≥ 5		*df*	*t*	*p*	*d*
	*M*	*SD*	*M*	*SD*				
Satisfaction with information received from oncological team	3.89	0.97	3.35	1.14	108	2.61	0.01	0.517
Threat perception regarding COVID-19 infection	3.39	1.04	3.61	1.14	118	−1.05	0.296	−0.202
Fear regarding potential cancer deterioration due to changed treatment plans	1.96	1.08	2.43	1.36	68.84	−2.06	0.059	−0.367

DT, Distress Thermometer; d, Cohen’s d effect size.

**Table 3:** Group differences for patients above and below the cut-off for symptoms of depression (PHQ-2).

Items	PHQ-2 < 3		PHQ-2 ≥ 3		*df*	*t*	*p*	*d*
	*M*	*SD*	*M*	*SD*				
Satisfaction with information received from oncological team	3.73	1.04	3.31	1.25	107	1.44	0.153	0.389
Threat perception regarding COVID-19 infection	3.35	1.05	4.31	0.95	117	−3.44	0.001	−0.925
Fear regarding potential cancer deterioration due to changed treatment plans	2.06	1.22	2.65	1.17	118	−1.84	0.068	−0.483

PHQ-2, Patient Health Questionnaire-2; *d*, Cohen’s d effect size.

**Table 4:** Group differences for patients above and below the cut-off for symptoms of anxiety (GAD-2).

Items	GAD-2 < 3		GAD-2 ≥ 3		*df*	*t*	*p*	*d*
	*M*	*SD*	*M*	*SD*				
Satisfaction with information received from oncological team	3.76	1.03	3.26	1.19	109	1.86	0.065	0.47
Threat perception regarding COVID-19 infection	3.44	1.09	3.72	1.02	119	−1.03	0.303	−0.264
Fear regarding potential cancer deterioration due to changed treatment plans	2.11	1.24	2.37	1.65	120	−0.86	0.394	−0.213

GAD-2, Generalized Anxiety Disorder-2; *d*, Cohen’s d effect size.

### 3.4. Determinants of distress

Results of the multiple linear regression model I revealed significant associations between psychosocial distress and fear of disease deterioration (*β* = 0.195, *p* = 0.040) and significant negative associations between distress and satisfaction with information (*β* = −0.232, *p* = 0.014).

Model II indicated that only somatic symptoms showed significant associations with the level of psychosocial distress reported (*β* = 0.475, *p* < 0.000). Nagelkerk’s *R*^2^ for the model II of the total sample showed an explained variance of = 0.380 (adjusted: 0.322), for details see Table 5.

**Table 5:** Multiple linear regression predicting psychosocial distress (*n* = 107).

Determinants	*Modell 1*				*Modell 2*			
	*B*	*SE B*	*β*	*p*	*B*	*SE B*	*β*	*p*
Constant								
**COVID-19-related stressors**								
Satisfaction with information received from oncological team	−0.520	0.208	−0.232	*0.014*	*−0.241*	*0.195*	*−0.107*	*0.221*
Threat perception regarding COVID-19 infection	0.260	0.216	0.112	*0.233*	*0.036*	*0.206*	*0.015*	*0.863*
Concerns regarding potential cancer deterioration due to changed treatment plans	0.377	0.181	0.195	*0.040*	*0.090*	*0.173*	*0.046*	*0.603*
**Sociodemographic, clinical, and psychological variables**								
Age					*−0.013*	*0.015*	*−0.073*	*0.385*
Sex								
Male	*Ref.*							
Female					*−0.251*	*0.431*	*−0.052*	*0.561*
Current treatment								
No	*Ref.*							
Yes					*0.511*	*0.547*	*0.082*	*0.352*
Somatic symptoms					*0.213*	*0.045*	*0.475*	*<0.001*
Previous or current psych. treatment								
No	*Ref.*							
Yes					*0.085*	*0.446*	*0.018*	*0.849*
Self-efficacy					*−0.390*	*0.288*	*−0.120*	*0.179*

Modell 1: *R*^2^ = 0.120 (adjusted *R*^2^ = 0.094); Modell 2: *R*^2^ = 0.380 (adjusted *R*^2^ = 0.322).

### 3.5. Determinants of symptoms of depression (PHQ-2)

In Model I significant negative associations were displayed between symptoms of depression and satisfaction with information (*β* = −0.206, *p* = 0.027) and significant associations with fear of disease deterioration (*β* = 0.235, *p* = 0.013).

Significant negative associations with depressive symptoms in model II were found with self-efficacy (*β* = −0.170, *p* = 0.049) and significant positive associations with somatic symptoms (*β* = 0.449, *p* < 0.000). The model II for the total sample showed an explained variance of Nagelkerk’s *R*^2^ = 0.420 (corrected: 0.367), see Table 6.

**Table 6:** Multiple linear regression predicting depression (*n* = 108).

Determinants	*Modell 1*				*Modell 2*			
	*B*	*SE B*	*β*	*p*	*B*	*SE B*	*β*	*p*
Constant								
**COVID-19-related stressors**								
Satisfaction with information received from oncological team	−0.270	0.120	−0.206	*0.027*	*−0.111*	*0.110*	*−0.085*	*0.315*
Threat perception regarding COVID-19 infection	0.175	0.126	0.129	*0.166*	*−0.013*	*0.117*	*−0.010*	*0.908*
Concerns regarding potential cancer deterioration due to changed treatment plans	0.264	0.104	0.235	*0.013*	*0.100*	*0.098*	*0.089*	*0.309*
**Sociodemographic, clinical, and psychological variables**								
Age					*0.005*	*0.008*	*0.047*	*0.565*
Sex								
Male	*Ref.*							
Female					*−0.072*	*0.245*	*−0.025*	*0.771*
Current treatment								
No	*Ref.*							
Yes					*0.249*	*0.311*	*0.067*	*0.426*
Somatic symptoms					*0.119*	*0.026*	*0.449*	*<0.001*
Previous or current psych. treatment								
No	*Ref.*							
Yes					*−0.255*	*0.254*	*−0.090*	*0.318*
Self-efficacy					*−0.324*	*0.163*	*−0.170*	*0.049*

Modell 1: *R*^2^ = 0.138 (adjusted *R*^2^ = 0.113); Modell 2: *R*^2^ = 0.420 (adjusted *R*^2^ = 0.367).

### 3.6. Determinants of symptoms of anxiety (GAD-2)

Model I of the multiple linear regression showed a significant negative association between anxiety symptoms and satisfaction with information (*β* = −0.347, *p* < 0.000).

Model II revealed that the negative association between anxiety and satisfaction with information received remained significant (*β* = −0.347, *p* < 0.000). In addition, a positive association with somatic symptoms emerged (*β* = 0.395, *p* < 0.000). Explained variance of this model was Nagelkerk’s *R*^2^ = 0.413 (corrected: 0.359), see Table 7.

**Table 7:** Multiple linear regression predicting anxiety (*n* = 108).

Determinants	*Modell 1*				*Modell 2*			
	*B*	*SE B*	*β*	*p*	*B*	*SE B*	*β*	*p*
Constant								
**COVID-19-related stressors**								
Satisfaction with information received from oncological team	−0.442	0.115	−0.347	*<0.001*	*−0.266*	*0.108*	*−0.208*	*0.016*
Threat perception regarding COVID-19 infection	0.091	0.120	0.069	*0.448*	*−0.111*	*0.114*	*−0.084*	*0.334*
Concerns regarding potential cancer deterioration due to changed treatment plans	0.183	0.099	0.168	*0.067*	*0.078*	*0.095*	*0.072*	*0.415*
**Sociodemographic, clinical, and psychological variables**								
Age					*0.006*	*0.008*	*0.061*	*0.456*
Sex								
Male	*Ref.*							
Female					*0.095*	*0.240*	*0.034*	*0.694*
Current treatment								
No	*Ref.*							
Yes					*0.543*	*0.304*	*0.151*	*0.078*
Somatic symptoms					*0.102*	*0.025*	*0.395*	*<0.001*
Previous or current psych. treatment								
No	*Ref.*							
Yes					*−0.275*	*0.160*	*−0.161*	*0.064*
Self-efficacy					*−0.299*	*0.160*	*−0.161*	*0.064*

Modell 1: *R*^2^ = 0.173 (adjusted *R*^2^ = 0.149); Modell 2: *R*^2^ = 0.413 (adjusted *R*^2^ = 0.359).

## 4. Discussion

This study investigated potential associations of COVID-19-related stressors, more specifically information satisfaction, threat perception and fear of disease deterioration with distress and symptoms of depression and anxiety in German out-patients with cancer during the second wave of the COVID-19 pandemic. Results revealed that initially, satisfaction with information was negatively associated with all three outcomes. Fear of disease deterioration was associated with distress and depressive symptoms. However, after controlling for sociodemographic, clinical and psychological characteristics, only satisfaction with information remained an independent determinant of anxiety. Patients who were less satisfied with the information they received from their oncological team regarding the impact of a COVID-19 infection on the disease reported higher anxiety. All three outcomes were most strongly determined by the level of somatic symptoms (highest standardized regression coefficient).

While several studies showed that tumor type or stage did not influence psychological symptomatology of cancer patients during the pandemic (Bartmann et al., 2021; Toquero et al., 2021; Obispo-Portero et al., 2022), it remained unclear whether physical symptom burden represents a risk factor. Here, for all three measures of psychosocial strains – distress, depression and anxiety – somatic symptoms were an independent predictor. These symptoms include pain, sleep disturbances or dizziness, which are well known to increase distress in cancer patients and deteriorate psychological wellbeing (Riedl and Schüßler, 2022). The association between physical and psychological symptoms has been reported previously and is well-established. For example, a study among cancer in-patients showed that a worse physical condition is associated with higher distress (Pichler et al., 2022b). Another study by Leonhart et al. (2017) revealed that higher somatic symptom severity is associated with higher levels of depression and anxiety as well as threatening and negative illness perceptions. This association could indicate both, that a higher symptom burden is a risk factor for distress or symptoms of depression or anxiety, or that patients, who are distressed, anxious or depressed might observe somatic symptoms more and assess them differently. A further explanation might be that cancer patients with a high somatic symptom burden rely more on the health care system than patients with few or no somatic symptoms and are therefore faced with pandemic-induced difficulties such as postponed treatments or shortage of certain medication. For clinical practice, special attention is required toward patients with high somatic symptom level as they might represent a particularly vulnerable group.

Several other studies showed that COVID-19-related stressors like changes in treatment plans, treatment disruption or delays, or worry about COVID-19, were predictors for oncology patients’ high levels of distress, anxiety and depressive symptoms (Chen et al., 2020; Bäuerle et al., 2021; Eckford et al., 2021; Gultekin et al., 2021). However, unlike our study, these investigations did not control for somatic symptom burden. So, our study tentatively suggests that physical well-being overrides the relevance of some COVID-19-related stressors for oncological patients’ psychological well-being. Patients’ physical symptoms might be associated with the cancer disease, with cancer treatments, or they might be unrelated to cancer but evoke fear of cancer coming back. Thus, it seems plausible that physical symptoms are strongly tied to personal wellbeing as they are associated with suffering from cancer, which might be more central to personal wellbeing than the possibility of getting infected with SARS-CoV-2. This corresponds with the results by Gultekin et al. (2021), who showed that only 17.5% of the surveyed patients with gynecological cancer indicated that they were more afraid of COVID-19 than of cancer.

However, satisfaction with information seems to be important beyond physical wellbeing, as this emerged as an independent determinant of anxiety. Given the various uncertainties caused by the pandemic, feelings of anxiety and fear are found to be dominant in COVID-19-related communications (Gharzai et al., 2020). The association between satisfaction with information and anxiety in cancer patients has been found in studies prior to the pandemic. Thus, it seems that our results confirm a common phenomenon, which also occurred during the COVID-19 pandemic. Similarly, Bäuerle et al. (2021) found that satisfaction with general information regarding COVID-19 is protective toward an increase of anxiety in cancer patients. Again, while our results could indicate that unsatisfactory communication could increase anxiety, they could also reflect that anxious, distressed or depressed patients are less satisfied with the communication.

Notably, patients’ satisfaction with information might not reflect the degree of information they actually received from their oncological team. Several patients mentioned that they had not talked with their physician about potential impacts of COVID-19 on their cancer disease in particular, but still stated being satisfied. Therefore, the item might indicate a more general satisfaction with communication and care, influenced by other variables such as feeling safe from COVID-19 by the undertaken protective measures or trusting their physician that relevant information is being transferred to patients.

Generally, in this study, psychosocial distress as well as depression and anxiety symptoms of oncological patients were not considerably elevated. About 35% of the cancer patients reported clinically elevated psychosocial distress which is comparable to data of distress among cancer patients before the COVID-19 pandemic (Mehnert et al., 2018). Within this study, symptoms of anxiety were elevated in 15.6% of participants, symptoms of depression in 14.2%. Inconsistent results have been published with regard to psychological symptoms during the pandemic: Bäuerle and colleagues found a significant increase in distress as well as depression and anxiety symptoms in cancer patients before as compared to after the outbreak of the pandemic (Bäuerle et al., 2021). A number of studies found increased levels of anxiety and depression in cancer patients as compared to the general population during the COVID-19 pandemic in 2020 (Toquero et al., 2021; Wong et al., 2021; Obispo-Portero et al., 2022). Other studies showed no difference in distress and anxiety between cancer patients and healthy controls during the pandemic (Musche et al., 2020; Bartmann et al., 2021).

Only a small percentage of the participants in our study indicated a strong fear of disease deterioration (4.9%). This might be accounted for by the fact that only 17.2% actually experienced treatment changes during the course of the pandemic. Besides, within this sample, 27% of participants were currently not undergoing any active treatment and only few COVID-19-related stressors were of high relevance to them. While many health care facilities faced challenges in delivering cancer care, especially during the beginning of the pandemic (Jazieh et al., 2020; Dinkel et al., 2021a), patients in this study were rarely affected by treatment changes and probably less afraid of (possible) disease deterioration. This might have also influenced their threat perception and their need for information: Maintaining regular treatment appointments and seeing one’s physician on a regular basis, might satisfy patients’ subjective need for information. Besides, fewer hospital visits due to canceled appointments would mean less risk of infection.

There was also a significant negative association between self-efficacy and depressive symptoms. Other studies have found that self-efficacy not only predicts depression but also mediates the connection between physical symptoms and depression (Rhondali et al., 2014). As a consequence, this might lead to more maladaptive and less active coping and further result in less perceived control (Nolen-Hoeksema and Aldao, 2011). Especially during a pandemic, self-efficacy could be limited, due to required protective measures (e.g., lockdown). In clinical practice, addressing potential sources of self-efficacy and active coping, even within limited options and uncertain circumstances, might help patients to increase their sense of control (Dijkstra and Homan, 2016).

The time of data collection should also be considered when interpreting the results displayed here. The data were collected during the “second pandemic wave” in Germany (11/2020–02/2021), almost 1 year after the first case of COVID-19. At this time, relevant experience that had been gained about how to deal with COVID-19 and important findings, e.g., about virus transmission and prevention, was already public knowledge. Furthermore, the distribution of the anti-COVID-vaccination began at the end of our data collection. A vaccination was therefore available to high risk patients within the foreseeable future.

### 4.1. Limitations

A strength of this study is its structured and comprehensive data collection within a short period of time, thus ensuring a stable context relating to the pandemic situation and public health measures taken. One limitation is that we did not include any comparison group, i.e., levels of distress, depression and anxiety within a healthy population during the same time period. Besides, the sample size was relatively small. Since consecutive sampling was applied and only patients from two university hospitals were included, the generalizability of the results is limited.

### 4.2. Conclusion and recommendations

While COVID-19-related stressors impact cancer patients’ psychological health, physical wellbeing seems to outweigh some of these effects. Physical symptoms are strongly associated to psychological wellbeing and might be more central to personal wellbeing than the possibility of getting infected with SARS-CoV-2. Receiving adequate information seems to be of importance beyond these associations. Maintaining medical care wherever possible and continuing communication during crises such as a pandemic can serve as stabilizing factors for cancer patients. Providing continuous, comprehensive information and transparency is crucial, especially during uncertain and challenging periods of a cancer disease (Pichler et al., 2021; Sokas et al., 2021). By making plans and discussing possible scenarios and adaptations, patients’ anxiety and psychosocial distress can be reduced.

## Data availability statement

The raw data supporting the conclusions of this article will be made available by the authors, without undue reservation.

## Ethics statement

The studies involving human participants were reviewed and approved by Ethics Committee of the Faculty of Medicine, Technical University of Munich, and Ethics Committee of the Medical Faculty, University of Munich (LMU). The patients/participants provided their written informed consent to participate in this study.

## Author contributions

AD, FM, TF, and TP designed the study. TF, TP, SM, IB, and TA obtained the data. NH, HA, VH, and KH provided administrate and technical support and supervision of data acquisition. TF, TP, and AD performed the statistical analyses. TF and TP wrote the first draft. All authors read the manuscript for critical revision. All authors contributed to the article and approved the final version.

## Conflict of interest

The authors declare that the research was conducted in the absence of any commercial or financial relationships that could be construed as a potential conflict of interest.

## Publisher’s note

All claims expressed in this article are solely those of the authors and do not necessarily represent those of their affiliated organizations, or those of the publisher, the editors and the reviewers. Any product that may be evaluated in this article, or claim that may be made by its manufacturer, is not guaranteed or endorsed by the publisher.
